# The Novel *Yersinia enterocolitica* Telomere Phage vB_YenS_P840 Is Closely Related to PY54, but Reveals Some Striking Differences

**DOI:** 10.3390/v15102019

**Published:** 2023-09-28

**Authors:** Julia Anabell Bräuer, Jens Andre Hammerl, Sabrin El-Mustapha, Julius Fuhrmann, Andrea Barac, Stefan Hertwig

**Affiliations:** German Federal Institute for Risk Assessment, Department of Biological Safety, Max-Dohrn Str. 8–10, D-10589 Berlin, Germanyjens-andre.hammerl@bfr.bund.de (J.A.H.);

**Keywords:** *Yersinia*, phage, telomere, plasmid prophage, regulation, PY54, N15

## Abstract

Telomere phages are a small group of temperate phages, whose prophages replicate as a linear plasmid with covalently closed ends. They have been isolated from some *Enterobacteriaceae* and from bacterial species living in aquatic environments. Phage PY54 was the first *Yersinia* (*Y.*) *enterocolitica* telomere phage isolated from a nonpathogenic O:5 strain, but recently a second telomeric *Yersinia* phage (vB_YenS_P840) was isolated from a tonsil of a wild boar in Germany. Both PY54 and vB_YenS_P840 (P840) have a siphoviridal morphology and a similar genome organization including the primary immunity region immB and telomere resolution site *telRL*. However, whereas PY54 only possesses one prophage repressor for the lysogenic cycle, vB_YenS_P840 encodes two. The *telRL* region of this phage was shown to be processed by the PY54 protelomerase under in vivo conditions, but unlike with PY54, a flanking inverted repeat was not required for processing. A further substantial difference between the phages is their host specificity. While PY54 infects *Y. enterocolitica* strains belonging to the serotypes O:5 and O:5,27, vB_YenS_P840 exclusively lyses O:3 strains. As the tail fiber and tail fiber assembly proteins of the phages differ significantly, we introduced the corresponding genes of vB_YenS_P840 by transposon mutagenesis into the PY54 genome and isolated several mutants that were able to infect both serotypes, O:5,27 and O:3.

## 1. Introduction

Most temperate phages integrate their genome into the bacterial chromosome when they enter the lysogenic cycle. However, some prophages replicate as circular or linear plasmids. Among the temperate phages replicating as linear plasmids during lysogeny, there is a small group of phages whose prophages possess terminal hairpin ends (telomeres). Thus far, eight telomere phages have been phenotypically and genetically characterized. The first described telomere phage was *E. coli* phage N15, which was discovered in 1964 in Moscow, Russia [1]. Some decades later, the telomere phages PY54 and phiKO2 were isolated from *Y. enterocolitica* and *Klebsiella oxytoca*, respectively [2,3]. In the following years, a number of telomere phages have been isolated from the marine species *Vibrio parahaemolyticus*, *Halomonas aquamarina* and *Aeromonas hydrophila* [4,5,6,7,8,9]. Even though enterobacterial and marine telomere phages have different morphologies and are only distantly related at the nucleotide level, they share a similar genome organization. Their genomes are composed of two arms, separated by a large palindromic sequence termed *telRL* [2,4,5,6,7,8,10,11]. This site is the substrate for the phage-encoded enzyme protelomerase TelN, whose gene is located immediately upstream of *telRL*. After injection of the phage DNA into the host cell, the phage genome circularizes by its cohesive ends, followed by the processing of *telRL* by the protelomerase. This enzyme has cleaving/joining activity and introduces staggered cuts into the palindromic sequence. The overhangs are self-complementary, fold back and are then joined by TelN resulting in the generation of the hairpin ends of the linear plasmid prophage, which is approximately 50% circularly permuted to the phage genome [12,13]. While the left arm of the phage genome mainly encodes proteins for virion assembly and plasmid partitioning, the right arm upstream of *telRL* and *telN* contains genes for plasmid replication, host cell lysis and the regulation of the lytic and lysogenic cycle. As with most other temperate phages, telomere phages possess a genetic switch. All of them contain a primary immunity region (immB), which is comparable to the immunity region of lambda-like phages, but exhibits a simpler arrangement. In the enterobacterial telomere phages N15, phiKO2 and PY54, immB encodes products related to the prophage repressor CI, lytic repressor Cro and a putative anti-terminator Q as well as operator sites located between *cI* and *cro* [10,14,15,16]. The genes *cro* and *q* of these phages are arranged in one operon. However, in the PY54 operon, *cro* and *q* are separated by an additional open reading frame (ORF42) whose function is unknown [14]. Interestingly, a *cro*-related gene could not be identified in any of the marine telomere phages. Instead, they contain an ORF at the position of *cro* whose product shows some relationship to the PY54 ORF42 product. For the marine telomere phage VP58.5, it was shown that this ORF encodes a second prophage repressor. In addition to immB, the enterobacterial telomere phages harbor the locus immA, which is an operon in N15 and phiKO2 comprising three genes coding for the repressor AntA, an inhibitor of cell division, Icd, and a protein denoted AntB whose function has not been elucidated as of yet. In PY54 an *antB* gene is missing, while *antA* and *icd* are widely separated on the right arm of the phage genome [2,7,10,11].

In this study, we describe the novel telomere phage vB_YenS_P840 isolated from a wild boar. Unlike PY54, vB_YenS_P840 exclusively infects *Y. enterocolitica* O:3 strains. To identify other differences between the two phages, we analyzed the activity of possible repressor genes, examined the processing of the vB_YenS_P840 *telRL* site by the PY54 protelomerase and demonstrated that the tail fiber genes are responsible for the diverse host specificity. In addition, we examined the activity of vB_YenS_P840 mutants harboring an antibiotic resistance gene and constructed a linear miniplasmid of the phage. This study revealed some significant discrepancies between these otherwise closely related phages.

## 2. Materials and Methods

### 2.1. Bacterial Strains and Culture Conditions

Relevant information about bacterial strains used in this study is given in Table 1. The strains originate from the culture collection of the Consiliary Laboratory for *Yersinia* (KL *Yersinia*) hosted at the German Federal Institute for Risk Assessment (BfR), Berlin, Germany. *Escherichia coli* (*E. coli*) and *Yersinia* spp. bacteria were cultivated in/on lysogeny broth (LB)-based media at 37 and 28 °C, respectively. The cultivation in a liquid medium was conducted under continuous shaking at 200 rpm [17]. If required, LB medium was supplemented with antibiotics at the following concentrations: 12.5 µg mL^−1^ ampicillin, 12.5 µg mL^−1^ chloramphenicol and 100 µg mL^−1^ kanamycin.

### 2.2. Isolation, Propagation, Purification and Host Range Determination of vB_YenS_P840

Phage vB_YenS_P840 was isolated from the tonsil of a wild boar in northeast Germany during the hunting season 2019/2020. Upon arrival of the cooled tonsil in the lab, five milliliters of SM buffer were added to the sample, which was then incubated on a stirrer at 4 °C overnight. Thereafter, the material was subjected to centrifugation for 20 min at 8000 rpm and 10 °C. The supernatant was passed through a 0.45 μm pore size filter (VWR International, Darmstadt, Germany) and stored until further use at 4 °C. The determination of lytic activity and the host range of the phage were performed by spotting 10 μL of a serial dilution of the sample onto a lawn of *Y. enterocolitica* indicator strains belonging to various bio-/serotypes [22]. For this, 100 μL of the respective indicator strain was mixed with 6 mL prewarmed NZCYM (VWR International, Darmstadt, Germany) soft agar (0.6%) and poured onto an LB agar plate [23]. After incubation overnight at RT, 28 °C and 37 °C, agar plates were inspected for plaque formation. Plaques were subjected to a three-fold plaque purification procedure. High-titer lysates of the phage were obtained by preparing 10–20 agar plates with confluent lysis of the *Y. enterocolitica* B4/O:3 strain 18-YE00024. The soft agar was harvested by scraping and resuspended in an SM buffer for several hours. Thereafter, the lysates were centrifuged for 20 min at 10,000× *g* to remove agar and debris and then filtered (see above). For purification, vB_YenS_P840 particles were concentrated by ultracentrifugation and applied onto a CsCl step gradient as previously described [22].

### 2.3. Transmission Electron Microscopy (TEM)

The CsCl-purified phage was investigated by TEM using the negative staining procedure with uranyl acetate (VWR International, Darmstadt, Germany) as previously described [24]. Specimens were examined by TEM using a JEM-1010 (JEOL, Tokyo, Japan) at 80 kV acceleration voltage.

### 2.4. Isolation of Phage DNA, Sequencing and Genome Annotation

Illumina short-read, paired-end whole-genome sequencing (WGS) was performed using phage DNA extracted from concentrated virions as previously described [23]. The DNA Flex Library Preparation kit (Illumina, San Diego, CA, USA) was used for sequencing library preparation, and WGS was conducted on an Illumina NextSeq500 benchtop device [25]. Raw reads were subjected to de novo assembling using the spades algorithm of the Pathosystems Resource Integration Center (PATRIC) database (version 3.6.20). For the prediction of putative coding sequences (CDS), the annotation tool of the PATRIC database was used. Further bioinformatics analysis (i.e., sequence comparison) was conducted using the blast-suite (blastn, blastx blastp; https://blast.ncbi.nlm.nih.gov/Blast.cgi, accessed in 2022/2023) of the National Center for Biotechnology Information (NCBI). The prediction of potential transcription terminators was conducted using ARNold [26,27]. If not otherwise indicated, default settings were used for the analyses. Dot plot illustrations were conducted using DS Gene (version 2.5; Accelrys Inc., San Diego, CA, USA) with parameters specified in the legends of the illustrations.

### 2.5. Isolation and Analysis of Phage Mutants

Cloramphenicol-resistant phage mutants were created by in vivo transposon mutagenesis using the mini-Tn5 transposon derivative mini-Tn5Cm [18,19]. The lysogenic *Y. enterocolitica* strain 18-YE00024 harboring vB_YenS_P840 was used as a recipient in a mating experiment with an *E. coli* K12 strain containing mini-Tn5Cm. Likewise, a kanamycin resistance gene was inserted into the strain described above by electroporation using the EZ-Tn5^TM^ Transposome Kit (Lucigen—LGC Bioresearch Technologies, Biozym Scientific GmbH, Hessisch Oldendorf, Germany). Transconjugants and transformants were selected on Cefsulodin Irgasan Novobiocin (CIN) agar supplemented with chloramphenicol and kanamycin, respectively. To isolate mutants in which the transposon had been inserted into the prophage, the transconjugants and transformants were pooled and prophages were induced by treatment with mitomycin C. The lysates obtained were used to lysogenize a susceptible *Y. enterocolitica* strain. Phage mutants were detected by plating the infected bacteria on agar containing chloramphenicol or kanamycin.

To determine the integration site of the resistance gene within the vB_YenS_P840 genome, restriction fragments of the mutants were ligated with the vector pMCS5, followed by electroporation of the strain *E. coli* GeneHogs and selection of transformants on agar plates containing the respective antibiotic. Thereafter, plasmids of transformants were isolated and sequenced using primers deduced from the resistance genes.

### 2.6. Molecular Cloning and Activity of vB_YenS_P840 Repressor Genes

To determine the influence of vB_YenS_P840 repressor genes on the lifestyle of phage PY54, *cI*, *cro*, *q*, *antA* and ORF40 (counterpart of ORF42 of PY54) were amplified by PCR using forward and reverse primers with embedded restriction sites for NdeI and HindIII, respectively (Supplementary Materials Table S1). Upon amplification and digestion, the products were ligated with the expression vector pMS470∆8 and introduced into *E. coli* GeneHogs. The resulting constructs pJB001 (*cI*), pJB002 (*cro*), pJB003 (ORF40), pJB004 (*antA*) and pJB010 (*q*) were then introduced into the *Y. enterocolitica* strain 83/88/2 by electroporation.

Lysis and lysogenization of the recombinant *Y. enterocolitica* strains as well as of control strains with or without the vector pMS470∆8 were determined using the phage PY54 mutant O containing a kanamycin resistance gene (Table 1). To examine the lytic effect of PY54, a double agar overlay assay was conducted. The evaluation of the lysogenic property of the phage on the plasmid-containing strains was performed on LB-agar plates supplemented with chloramphenicol and kanamycin, whereas lysogenization of the strain 83/88/2 devoid of a plasmid was determined on LB-agar plates supplemented only with kanamycin.

### 2.7. Resolution of the vB_YenS_P840 telRL Region by the PY54 Protelomerase

The vB_YenS_P840 *telRL* region including and excluding the flanking inverted repeat were amplified by PCR using primers with embedded restriction sites for HindIII and BamHI, respectively. The 182 bp *palIR* and 162 bp *palIRw* PCR products were ligated with the vector pIV2 and introduced into *E. coli* GeneHogs yielding the recombinant plasmids pJB005 and pJB006, respectively, which then were introduced into *Y. enterocolitica* strain 83/88/2 by electroporation.

In order to determine if PY54 can process the *telRL* site of vB_YenS_P840, *Y. enterocolitica* 83/88/2 strains harboring the constructs pJB005 and pJB006 were examined. Two 83/88/2 control strains, one containing the vector pIV2 and the other containing the construct pSH105 (pBR329 with the PY54 188 bp *palIR* sequence) were also analyzed. The amount of 7.5 mL LB-broth supplemented with an antibiotic (kanamycin for pIV2 or chloramphenicol for pBR329) was inoculated with 50 µL of the respective overnight culture. Once an optical density of 0.2 was reached, a 1.5 mL sample was taken and 300 µL of a lysate of PY54 mutant C (10^9^ pfu/mL, Table 1) was added to each strain. Further samples were taken after 10, 30, 60 and 120 min. The cells were pelleted (14,000 rpm for 5 min), the supernatant was removed and the pellet was stored in a freezer until further preparation. Plasmid isolation was then performed using the Invisorb^®®^ Spin Plasmid Mini Two kit (Stratec molecular, Germany). The processing of the plasmids was evaluated by agarose gel electrophoresis.

### 2.8. Insertion of vB_YenS_P840 Tail Fiber Genes into the PY54 Genome

To insert the vB_YenS_P840 tail fiber protein gene (ORF24) and tail fiber assembly protein gene (ORF25) into PY54 by in vivo transposon mutagenesis, the transposon delivery vector pUTKm [18,19] was modified by introducing a multiple cloning site and a P*tac* promoter into the plasmid. The vB_YenS_P840 tail fiber genes were amplified by PCR using a forward and reverse primer with an embedded restriction site for ApaI and NcoI, respectively. The 2.4 kb PCR product was ligated with the vector pMCS5. Upon transformation of the *E. coli* strain GeneHogs, recombinant clones were isolated and sequenced using vector primers and primers deduced from the tail fiber genes. A construct containing an insert without any sequence errors was digested with ApaI and NcoI and then ligated with pUTKm-MCS-TAC cleaved by the same enzymes resulting in pJB008. This construct was introduced into the *E. coli* strain S17.1 λpir [18,19] by electroporation. A transformant containing pJB008 and a *Y. enterocolitica* strain 83/88/2 containing the PY54 wildtype plasmid prophage were used for filter mating overnight at 28 °C. Thereafter, the bacteria were resuspended in 1 mL of SM buffer and plated on CIN-agar plates supplemented with kanamycin. After incubation for 24 h at 28 °C, transconjugants were pooled. The pool was transferred to a 200 mL LB medium supplemented with kanamycin, and prophages were induced by exposure to mitomycin C (1 mg/mL) after the cells reached an OD of 0.2. The cell suspension was incubated overnight and centrifuged at 10,000 rpm for 30 min at 4 °C, followed by filtration of the supernatant with a 0.22 µm bottle top filter (Corning Life Sciences).

PY54 mutants containing the kanamycin resistance gene and the vB_YenS_P840 tail fiber genes were identified by a lysogenization experiment. For this, 100 µL of the strains 83/88/2 and 18-YE00024 were infected with 300 µL of the phage lysate and plated on agar supplemented with kanamycin. As controls, both strains and the phage lysate were plated separately. After incubation overnight at 28 °C, the success of the transposon mutagenesis was assessed.

### 2.9. Nucleotide sequence accession

The genome of *Y. enterocolitica* phage vB_YenS_P840 was deposited at GenBank under the accession number OQ716682. The sequence of the transposon cloning vector pUTKm-MCS-Tac (Amp^R^, Km^R^) is available under the accession number OQ474598.

## 3. Results

### 3.1. vB_YenS_P840 and PY54 Are Closely Related Telomere Phages

**Figure 1:**
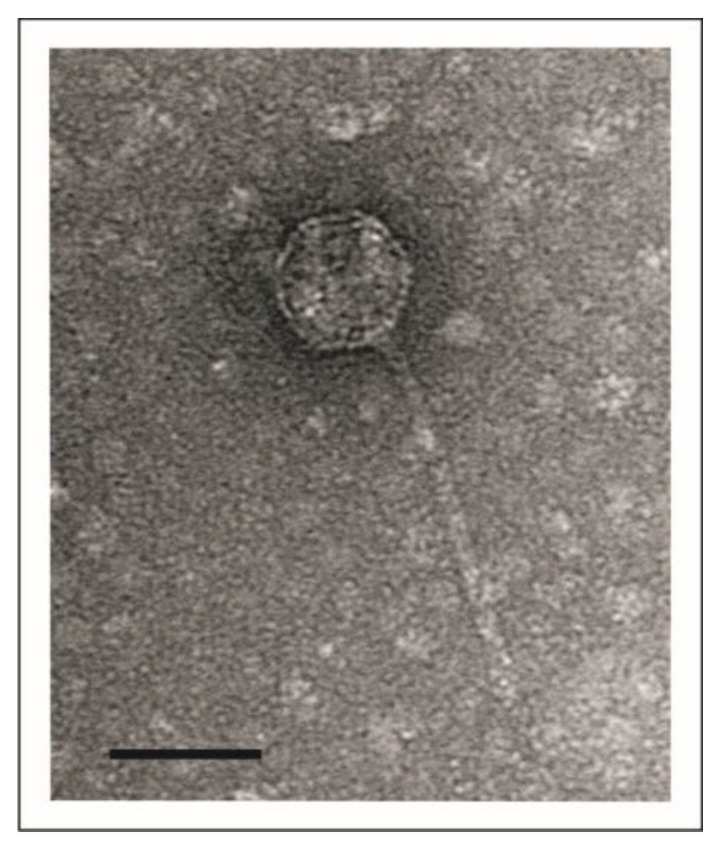
Transmission electron micrograph of vB_YenS_P840. The black bar represents a size of 50 nm.

The enterobacterial telomere phages described thus far have a siphoviridal morphology. Electron microscopic analysis of vB_YenS_P840 particles showed that this phage also belongs to this group (Figure 1).

**Figure 2:**
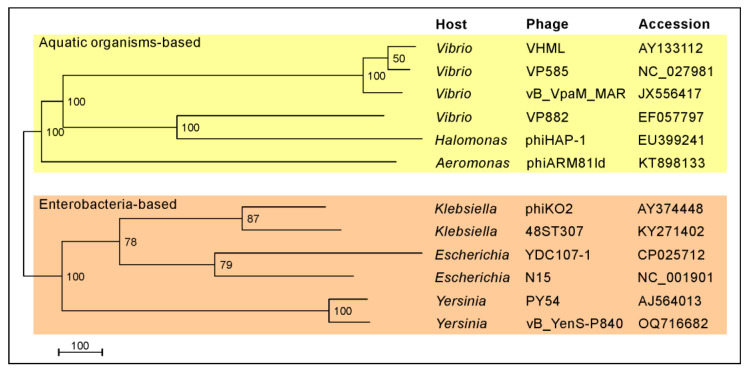
Relationship of telomere phages that have been sequenced to date. Phylogenomic GBDP (Genome-BLAST Distance Phylogeny) tree inferred using the formula D0 and yielding an average support of 88%. The numbers above the branches are GBDP pseudo-bootstrap support values from 100 replications. The branch lengths of the resulting VICTOR trees are scaled in terms of the respective distance formula used. The OPTSIL clustering yielded twelve species, four genera and one family cluster [29].

**Figure 3:**
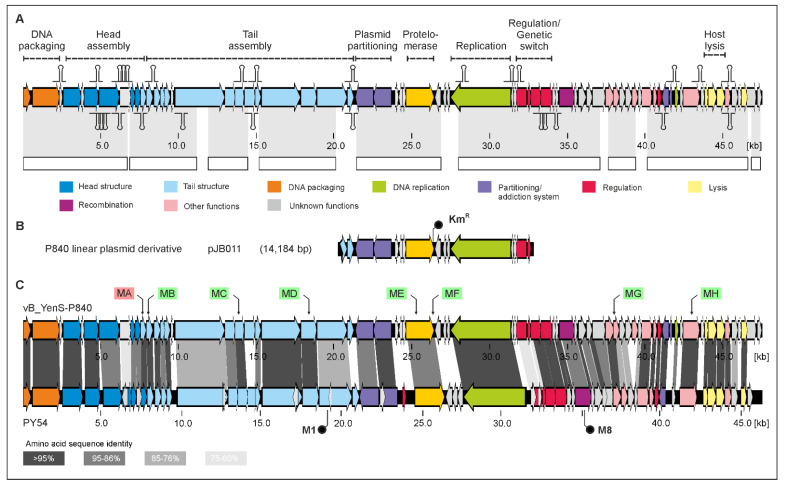
Gene map of phage vB_YenS_P840 and its relationship to PY54. (**A**) Gene map of vB_YenS_P840. Transcription terminators are given in Supplementary Materials, Table S2. White bars show regions of high nucleotide similarity (>75%) to *Yersinia enterocolitica* phage PY54 (coverage 86%, e-value 0.0, 89.95% identity, accession AJ564013.1). (**B**) Genes present in the linear miniplasmid are presented below. (**C**) Comparison of the vB_YenS_P840 and PY54 genomes. The positions of integrated resistance genes in the P840 genome are indicated by red (defective mutant) and green (intact mutants) boxes. The positions of the P840 tail fiber genes in the PY54 genome are indicated by black dots (see text for details).

It has an isometric head and a long, non-contractile tail, very similar to PY54. However, these two *Y. enterocolitica* phages exhibit completely different host ranges. Unlike PY54, which was isolated from a serotype O:5 strain and can additionally infect the serotype O:5,27, vB_YenS_P840 was shown to lyse exclusively O:3 strains after testing 50 *Y. enterocolitica* strains, 30 strains belonging to other *Yersinia* species as well as various other genera of Enterobacterales, e.g., *Escherichia*, *Klebsiella* and *Salmonella* [22,28]. Six out of ten O:3 strains were lysed by the phage (data not available). The highest lytic activity occurred at room temperature, whereas no lysis was observed at 37 °C. Sequence analysis of the phage genome revealed a size of 47,467 bp with the same 3’-protruding ends of ten nucleotides, like in PY54 [3]. The vB_YenS_P840 phage genome (full taxonomic lineage: viruses; Duplodnaviria; Heunggongvirae; Uroviricota; Caudoviricetes; unclassified Caudoviricetes) is 96.83% identical to that of PY54 and more distantly related to N15 and phiKO2 (Figure 2). Thus, it is not surprising that the two *Yersinia* phages share a very similar genome organization (Figure 3).

For vB_YenS_P840, whose genome is 1,128 bp larger than that of PY54, 72 open reading frames (ORFs) have been predicted (Supplementary Materials, Table S3), five more than for PY54, all of them encoding hypothetical proteins. To insert a marker into the vB_YenS_P840 genome, which is helpful for verifying lysogenization, identifying genes essential for the lytic or lysogenic cycle and constructing mini-plasmid derivatives of the prophage, a kanamycin and a chloramphenicol resistance gene were introduced into the prophage by transposon mutagenesis (see Section 2). Upon isolation of mutants, these were DNA-sequenced, and the insertion site of the respective resistance gene was determined. Eight different vB_YenS_P840 mutants (MA to MH) were isolated (Figure 3, Table 2).

Only mutant MA had lost the ability to lyse and lysogenize the host strain. In this mutant, the kanamycin resistance gene was integrated into ORF11 coding for a tail completion protein. By contrast, insertions within genes, which may encode other tail proteins (ORF12, 19 and 23) as well as genes for the protelomerase (ORF31), for an enzyme probably involved in DNA replication (ORF50) and for an adenine methylase (ORF62) did not prevent lysis or lysogeny. To construct a replicative linear miniplasmid, mutant F was selected, which harbored the kanamycin resistance intergenically just upstream of *telN*. The mutant was digested with the restriction endonucleases AflII and KpnI resulting in a fragment of 10,080 bp comprising ORF29 to 39 and the kanamycin resistance gene. After filling in the cohesive ends, the digest was treated with T4 ligase and electroporated into *E. coli*. The smallest plasmid, which was isolated from the transformants, had a size of 14,184 bp and was termed pJB011. It contained the fragment described above plus the adjacent 4.1 kb AflII/KpnI fragment containing ORF25 to 28 (Figure 3). To ascertain, whether this plasmid has a linear conformation, it was digested with several restriction enzymes, which cleave the plasmid once (HindIII) or twice (EcoRI and SalI). When cut once, a circular plasmid would show a single band on the agarose gel, whereas two bands would appear when the plasmid is cut twice. Supplemental Material Figure S1 shows that the digest with HindIII yielded two fragments and that with EcoRI or SalI three. Moreover, mapping of the restriction fragments clearly indicated that the ends of the plasmid were the result of *telRL* processing.

### 3.2. Phage vB_YenS_P840 Encodes Two Prophage Repressors and Three Proteins Stimulating the Lytic Cycle

**Figure 4:**
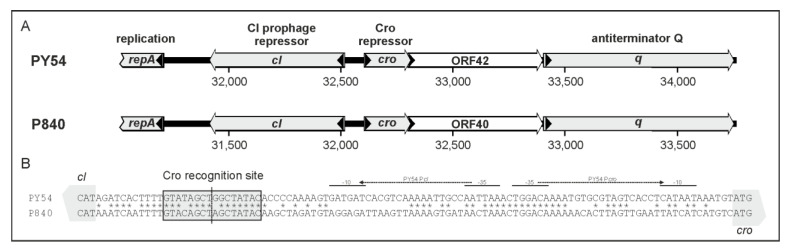
The immB regions of vB_YenS_P840 and PY54. (**A**) Organization of immB. (**B**) Intergenic region between *cI* and *cro*. The Cro recognition site and promoters identified in PY54 are indicated [14]. * indicates nucleotides conserved in the intergenic regions of PY54 and vB_YenS_P840.

The phages PY54 and vB_YenS_P840 possess a similar immB region, but an *icd* gene located in the left arm of the PY54 genome, which belongs to immA, is lacking in vB_YenS_P840 (Figure 3 and Figure 4).

To answer the questions of whether the putative vB_YenS_P840 repressors exert the same activities as their counterparts in PY54 and whether they influence also the lytic and lysogenic properties of this phage, the respective vB_YenS_P840 genes *cI*, *cro*, *q, antA* and ORF40, which corresponds to ORF42 of PY54, were introduced into the *Y. enterocolitica* O:5,27 strain 83/88/2. Following this, the transformants were infected by a PY54 mutant O containing a kanamycin resistance gene (mutant O, [10]). Lysis by PY54 was detected with all tested *Y. enterocolitica* isolates harboring a construct, the used vector or no plasmid (Table 3). However, the strongest lysis was observed with the constructs containing *cro, q* and *antA*, whereas plaques found in the bacterial lawn of the strains harboring *cI* or ORF40 were more turbid (data not available). On the other hand, the latter two genes resulted in a significant increase (almost two orders of magnitude) of lysogeny compared to the controls, while *cro, q* and *antA* completely prevented lysogenization by PY54. These results demonstrated that vB_YenS_P840 possesses two genes (*cI* and ORF40) encoding proteins with prophage repressor activity and three genes (*cro*, *q* and *antA*) acting as antagonistic repressors.

### 3.3. The vB_YenS_P840 telRL Site without Its Flanking Inverted Repeat Is a Substrate for the PY54 Protelomerase In Vivo

**Figure 5:**
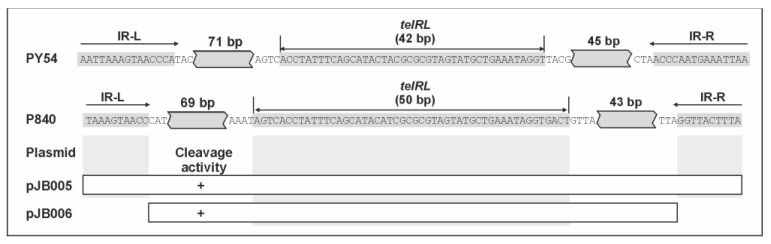
The *telRL* region of vB_YenS_P840 and PY54. The activity of the PY54 protelomerase on vB_YenS_P840 constructs is indicated.

Comparison of the very similar PY54 and vB_YenS_P840 *telRL* regions reveals two striking differences, (i) *telRL* is 8 bp longer than *telRL* of PY54, and (ii) the 15 bp inverted repeat flanking the *telRL* site, which has been suggested to be important for processing of PY54 *telRL* under in vivo conditions, is five nucleotides shorter in vB_YenS_P840 *telRL* (Figure 5).

**Figure 6:**
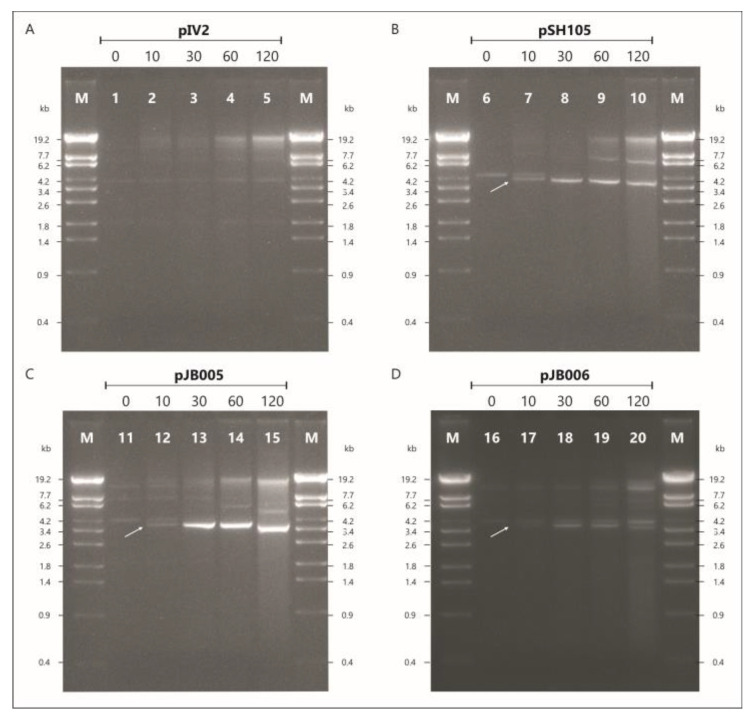
Telomere resolution of pSH105, pJB005 and pJB006 by the PY54 protelomerase. M λ StyI DNA marker. (**A**) Lanes 1 to 5, pIV2 (control); (**B**) lanes 6 to 10, pSH105 (PY54 *palIR*); (**C**) lanes 11 to 15, pJB005 (P840 *palIR*), (**D**) lanes 16-20, pJB006 (P840 *palIRw*). Processing was analyzed at the time point 0 and 10, 30, 60 and 120 min after adding the PY54 mutant M4/7. Arrows indicate processed plasmid bands.

We therefore first examined whether the *telRL* region of vB_YenS_P840 *telRL* is a substrate for the PY54 protelomerase in *Y. enterocolitica* 83/88/2 after infection with the phage. The experiment was conducted with plasmid pJB005 containing the complete vB_YenS_P840 *telRL* region, 182 bp in size (Figure 5). The vector pIV2 (Km^R^) [21] used for molecular cloning served as a negative control, whereas the construct pSH105 containing the PY54 *telRL* region (188 bp) was used as a positive control [3]. PY54 mutant C (M4/7) was utilized for this procedure due to the fact that the insertion of a kanamycin resistance gene within *cro* rendered the phage unable to enter the lytic cycle, leading to an exclusive lysogenization of the host upon infection [10]. The results of the gel electrophoretic analysis are depicted in Figure 6.

Before infecting the cells, the pSH105 and pJB005 plasmid profiles were identical to the one of the negative control. No conversion of the pIV2 vector was detected throughout the experiment (Figure 6, lanes 1–5). A proportional increase in the amount of plasmid by time due to bacterial growth was observed. On the contrary, for the positive control, pSH105 containing the PY54 *telRL* region (Figure 6, lane 6–10) plasmid processing was already visible 10 min after infection. This was discernible by the second DNA band that manifested at approximately 4 kb, right below the linearized plasmid (indicated by an arrow). This DNA band did not appear in the negative control. Thirty min upon infection, most of the plasmid DNA was processed by the protelomerase. The same could be seen with pJB005 containing the vB_YenS_P840 *telRL* region (Figure 6, lane 11–15). This clearly indicated that the *telRL* region of this phage including the 10 bp inverted repeat was also processed by the PY54 protelomerase. We then wanted to find out if the flanking inverted repeat was required for processing. Therefore, a DNA fragment containing the *telRL* region devoid of the flanking inverted repeat was introduced into *Yersinia* and tested. In this experiment, pJB005 functioned as a positive control, whereas pIV2 remained the negative control. pIV2 was again not processed at any time during the experiment (data not available). On the other hand, plasmid pJB006 without the inverted repeat showed a similar pattern to the positive control pJB005 (Figure 6, lane 16–20). Starting at 10 min, two linear plasmid conformations could be detected in these samples. The lower band intensified after 30 and 60 min of incubation. The finding that the upper band did not completely disappear can be explained by the fact that here the titer of the added PY54 mutant was significantly lower than in the first experiment, resulting in a strongly decreased multiplicity of infection. Nevertheless, from these results, it can be concluded that the flanking inverted repeat is not required for the processing of the P840 *telRL* site by the PY54 protelomerase in vivo and thus is not necessary for telomere resolution.

### 3.4. Two Tail Fiber Proteins of vB_YenS_P840 Are Sufficient to Change the PY54 Host Range

**Figure 7:**
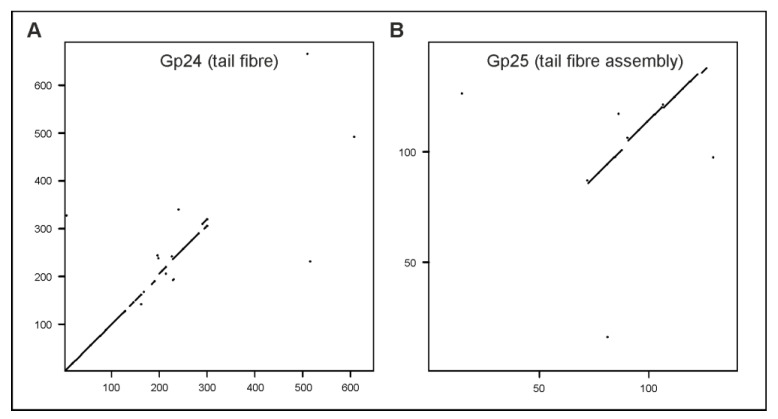
Dot plots of the vB_YenS_P840 and PY54 tail fiber (**A**) and tail fiber assembly protein (**B**).

One striking difference between the phages vB_YenS_P840 and PY54 is their host specificity. While PY54 only infects *Y. enterocolitica* strains belonging to the serotypes O:5 and O:5,27, vB_YenS_P840 exclusively lyses O:3 strains. Both phage genomes contain three genes (ORF23, 24 and 25 in vB_YenS_P840) whose products are related to proteins probably involved in tail fiber assembly. However, while ORF23 is 99% identical to PY54 ORF24, the products of ORF24 and ORF25, which may encode a tail fiber and tail fiber assembly protein, respectively, are more distantly related to their PY54 counterparts (Figure 7).

**Figure 8:**
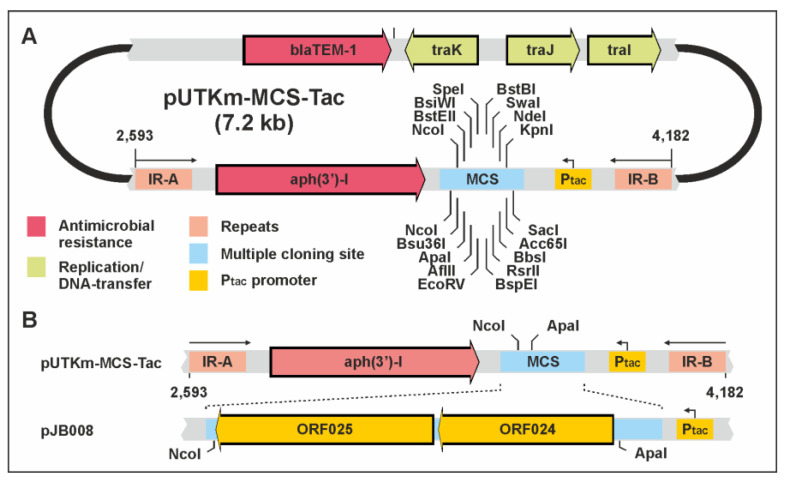
The newly constructed vector pUTKm-MCS-Tac (accession no. OQ474598) used for the insertion of the vB_YenS_P840 tail fiber genes into the PY54 genome. (**A**) Overview of the vector showing all relevant genetic elements. (**B**) Detail enlargement of the region between the inverted repeat and molecular cloning of the vB_YenS_P840 ORFs 24 and 25.

Thus, the question arises, whether these two ORFs determine the host specificity of vB_YenS_P840. To answer the question, we inserted both ORFs (2.4 kb) into the PY54 genome by transposon mutagenesis. To accomplish this, we introduced a multiple cloning site (MCS) and a *tac* promotor (P_tac_) into the transposon mutagenesis vector pUTKm [18,19], adjacent to the kanamycin resistance gene (Figure 8).

Upon PCR amplification of ORF24 and 25, both genes were inserted into the MCS, followed by the transformation of a suitable *E. coli* strain with this construct. The transformant was used as a donor in a mating experiment with the lysogenized *Y. enterocolitica* B2/O:5,27 strain 83/88/2 harboring PY54. The resulting transconjugants were then pooled and treated with mitomycin C, and the released phages were examined in terms of their ability to lysogenize strain 83/88/2. Ten colonies grew on agar containing kanamycin, whereas no growth was observed on plates on which strain 83/88/2 or the lysate was plated (data not available). We then induced the prophages in those ten colonies and studied the lysogenization of both the O:5,27 strain 83/88/2 and the B4/O:3 strain 18-YE00024, which served as hosts for vB_YenS_P840. Two mutants (M1 and M8, Figure 3) were able to lysogenize both strains. Sequence analysis revealed that the PY54 mutants indeed contained the vB_YenS_P840 ORFs 24 and 25 as well as the kanamycin resistance gene (*aph*(3**′**)-I). In mutant M1, this cluster of genes was integrated within the tail assembly protein gene (ORF27) of PY54 and in M8 within ORF46 probably coding for an exonuclease recombination-associated protein (Figure 3). Since both mutant phages were able to lyse both strains, it can be concluded that no essential function of PY54 was affected by the introduction of foreign genes.

## 4. Discussion

Telomere phages have yet only been isolated from some species belonging to the *Enterobacteriacae*, which are mostly associated with the gastrointestinal system of humans and animals, and from marine bacteria. Despite the diverse habitats, the different morphology and also the distant relationship at the nucleotide level, all enterobacterial and marine telomere phages described thus far exhibit a similar genome organization [14]. This also pertains to phage vB_YenS_P840, which was not isolated from a lysogenic strain by prophage induction but from the tonsil of a wild boar. The phage is closely related to the *Y. enterocolitica* telomere phage PY54, isolated twenty years ago [3]. However, some properties of these two phages differ significantly. First and foremost, they have a quite different host range. A closer look at the ORFs of the vB_YenS_P840 genome revealed two tail encoding genes (ORF24 and 25) whose products are significantly less related to the respective PY54 proteins than all other tail proteins of vB_YenS_P840. As these two genes probably code for a tail fiber protein and a tail fiber assembly protein, it is likely that they may determine the host specificity, as already shown for tail fiber proteins of other (*Yersinia*) phages [30,31,32,33]. We wanted to introduce these genes into the PY54 genome to verify their role, but we did not know at which position the genes could be inserted to avoid inactivation of essential functions and whether the phage has the capacity to encapsidate a larger genome, than its own. We therefore modified the transposon mutagenesis vector pUTKm [18,19] to facilitate an integration of the genes at random sites of the phage genome and identify lysogens by selection on agar plates containing kanamycin. Using this approach, two mutants were isolated exhibiting an extended host range. It is noteworthy that in one mutant (M1), the foreign genes were integrated into the gene for the tail fiber assembly protein, whose inactivation did not abolish the ability of the mutant to infect the serotype O:5,27. Thus, it can be suggested that the tail fiber assembly protein genes of phage PY54 and vB_YenS_P840 are interchangeable. Mutant 8 contained the insertion in a gene for an exonuclease recombination-associated protein, which is obviously not essential for the infection of the host strain. Moreover, the experiment indicated that PY54 can at least encapsidate DNA of 50.8 kb, approximately 10% more than its own genome.

The analysis of vB_YenS_P840 mutants revealed one gene (ORF11) for a putative tail completion protein whose inactivation resulted in a loss of phage activity, whereas some other tail protein genes (ORF12, 19, 23) are apparently dispensable for phage infection. Among them is a second predicted tail fiber protein encoded by ORF23. However, it cannot be excluded that these genes retained their activity, even though the insertions of the resistance gene occurred rather centrally, and not at one end of the respective ORF. Other insertions not affecting the lytic or lysogenic cycle were detected in ORF50 and ORF62 possibly encoding a helicase, polymerase or exonuclease and an adenine methylase, respectively. While the size of the predicted ORF50 product (approximately 12 kDa) might be too small for such an enzyme, the activity of an adenine methylase is probably not mandatory for phage infection. Interestingly, one mutant was isolated that showed an insertion within ORF31 encoding the protelomerase TelN, which also retained its activity. For circular plasmid replication, only *repA* is required [34]. We have not yet studied the conformation of the plasmid prophage of this mutant, but it is likely that it is a circular molecule. The linear conformation of the vB_YenS_P840 plasmid was demonstrated by the construction of a miniplasmid of 14.4 kb containing genes for plasmid partitioning, TelN, the replication protein RepA and the repressors CI and Cro. As the genes *telN* and *repA* and the telomere resolution region are the only elements required for the linear plasmid replication [34,35,36], it is likely that the size of the linear vB_YenS_P840 miniplasmid can be further reduced.

For PY54, it has been suggested that the 15 bp inverted repeat flanking *telRL* might be important for the processing of the site under in vivo conditions [3]. We therefore studied two vB_YenS_P840 constructs with and without the inverted repeat, which has only a length of 10 bp in this phage, regarding processing by the PY54 protelomerase. Since both constructs were processed, the additional inverted repeat in vB_YenS_P840 is not required. However, this does not of course mean that the 15 bp inverted repeat of PY54 is dispensable for processing. To answer this issue, further constructs of both phages should be examined.

The immB regions of vB_YenS_P840 and PY54 are very similar structurally and in terms of their encoded proteins. While for PY54 the Cro repressor binding site was narrowed down to a 16 bp sequence [14], the binding sites of the prophage repressor are still unknown. As shown in Figure 4B, the 16 nucleotides of the PY54 Cro binding site are similarly present in vB_YenS_P840, while other parts of the intergenic regions between the counter-orientated repressor genes are more distantly related. Thus, the results that the genes *cI*, *cro* and *q* of vB_YenS_P840 influenced the lytic and lysogenic cycle of PY54 could be expected. The same holds true for *antA*. However, surprisingly, vB_YenS_P840 ORF40, whose counterpart in PY54 is ORF42, exhibited a clear prophage repressor activity, whereas the corresponding ORF of PY54 did not influence the lifestyle of the phage [14]. The comparison of both proteins revealed that they are almost identical, but that the ORF42 product has an extension of 26 amino acids at the N-terminus (Figure 4A). Presumably, this extension resulted in the loss of activity of the PY54 protein. Interestingly, an ORF42 homolog does not exist in the enterobacterial telomere phages N15 and phiKO2 but was detected in the marine telomere phage VP58.5, where it also showed prophage repressor activity, even though the corresponding VP58.5 protein is only 32% identical to that of vB_YenS_P840 [14].

## 5. Conclusions

In conclusion, vB_YenS_P840 and PY54 are very similar phages that presumably share a common ancestor but diverged over time. The main discrepancies concern the genetic switch regulating the lytic and lysogenic cycle and a gene that determines the host specificity. Adaptations to a new host could be the driving force behind the observed changes.

## Figures and Tables

**Table 1:** Bacterial strains, phages and plasmids used in this study.

Reference (Accession No.)	Description/Genotype	Antimicrobial Resistance Phenotype	Strains (Plasmid/Vector) ^1^
Invitrogen	F^-^ *mcrA* ∆(*mrr*-*hsdRMS*-*mcrBC*) φ80*lacZ*∆M15 ∆*lacX*74 *recA*1 *araD*139 ∆(ara-leu)7697 *galU galK rpsL* (Str^R^) *endA*1 *nupG fhuA*::IS2 (confers phage T1 resistance)	Str^R^	*E. coli* GeneHogs
[18,19]	*S*train lysogenized with λpir phage containing the Tn5-based delivery plasmid pUTKm	Amp^R^, Km^R^	*E. coli* S17.1 λpir (pUTKm)
[20]	Bioserotype B2/O:5,27 strain lacking the virulence plasmid pYV		*Y. enterocolitica* 83/88/2
This work	Bioserotype B4/O:3 strain containing the vB_YenS_P840 (P840) prophage		*Y. enterocolitica* 18-YE00024
**Reference (Accession no.)**	**Description**	**Antimicrobial resistance**	**Phages**
This work (OQ716682)	P840 wildtype phage	None	vB_YenS_P840
[3,10]	PY54 wildtype phage	None	PY54
[3,10]	Lytic deficient PY54 transposon mutant	Km^R^	PY54 mutant C
[3,10]	Wildtype-like PY54 transposon mutant	Km^R^	PY54-mutant O
This work	Wildtype-like P840 transposon mutant	Km^R^	PY54 mutant M1 (*tfp*)
This work	Wildtype-like P840 transposon mutant	Km^R^	PY54 mutant M8 (*tfp*)
**Reference (Accession no.)**	**Description**	**Antimicrobial resistance**	**Plasmid/vector**
[18,19]	*E. coli* suicide mutagenesis vector	Amp^R^, Km^R^	pUTKm
This work (OQ474598)	pUTKm containing a MCS and P*tac* promoter	Amp^R^, Km^R^	pUTKm-MCS-Tac
This work	pUTKm-MCS-Tac with P840 ORF24 and 25 (ApaI/NcoI)	Amp^R^, Km^R^	pJB008
[14]	*E. coli* cloning vector	Amp^R^, Cat^R^	pMS470∆8*cat*
This work	pMS470∆8*cat* with P840 *cI* (NdeI/HindIII)	Amp^R^, Cat^R^	pJB001
This work	pMS470∆8*cat* with P840 *cro* (NdeI/HindIII)	Amp^R^, Cat^R^	pJB002
This work	pMS470∆8*cat* with P840 ORF40 (NdeI/HindIII)	Amp^R^, Cat^R^	pJB003
This work	pMS470∆8*cat* with P840 *antA* (NdeI/HindIII)	Amp^R^, Cat^R^	pJB004
This work	pMS470∆8*cat* with P840 *q* (NdeI/SphI)	Amp^R^, Cat^R^	pJB010
[21]	*Yersinia* cloning vector	Km^R^	pIV2
This work	pIV2 with P840 *palIR* (HindIII)	Km^R^	pJB005
This work	pIV2 with *P840 palIRw* (BamHI)	Km^R^	pJB006
MoBiTec, Göttingen	*E. coli* cloning vector	Amp^R^	pMCS5
This work	pMCS5 with P840 ORF 24 and 25 (ApaI/NcoI)	Amp^R^	pJB007
This work	vB_YenS_P840 mutant MF-derived miniplasmid (AflII/KanI)	Km^R^	pJB011
[3]	pBR329 with PY54 *palIR* (BamHI/HindIII)	Amp^R^, Cat^R^	pSH105

^1^ Additional *Yersinia* sp. isolates were used for host range determination as previously described ([22]); Abbreviations: Streptomycin (Str^R^), Ampicillin (Amp^R^), Chloramphenicol (Cat^R^) and Kanamycin (Km^R^).

**Table 2:** Lifestyle of vB_YenS_P840 mutants generated by in vitro transposon mutagenesis.

Lysogenization	Lytic Behaviour	Affected Gene	Resistance Marker Position	Mutant ID
−	−	ORF11 (tail completion protein)	7768 [Km^R^]	MA
+	+	ORF12 (tail protein)	8208 [Cat^R^]	MB
+	+	ORF19 (phage minor tail component)	13,866 [Cat^R^]	MC
+	+	ORF23 (tail fiber protein 1)	18,593 [Km^R^]	MD
+	+	ORF31 (TelN)	25,125 [Km^R^]	ME
+	+	intergenic (between *telN* ORF31 and ORF32)	26,449 [Km^R^]	MF
+	+	ORF50 (helicase, polymerase, exon.)	38,159 [Cat^R^]	MG
+	+	ORF62 (adenine methylase)	42,479 [Cat^R^]	MH

**Table 3:** Influence of vB_YenS_P840 repressors on the lifestyle of PY54.

Lysis (pfu/mL)	Lysogenization (cfu/mL)	vB_YenS_P840 Insert	Construct in *Y. enterocolitica* 83/88/2
5.27 × 10^6^	1.20 × 10^6^	*cI* (ORF38)	pJB001
9.21 × 10^6^	-	*cro* (ORF39)	pJB002
4.71 × 10^6^	2.55 × 10^6^	PY54-ORF42 (ORF40)	pJB003
6.76 × 10^6^	-	*antA* (ORF57)	pJB004
7.0 × 10^6^	-	*q* (ORF41)	pJB010
4.90 × 10^6^	3.54 × 10^4^	-	None
4.71 × 10^6^	4.29 × 10^3^	None	pMS470Δ8*cat*

## Data Availability

The whole genome sequence of the phage genome was submitted to GenBank.

## Supplementary Materials

The following supporting information can be downloaded at: https://www.mdpi.com/article/10.3390/v15102019/s1, Table S1. Primer sequences used for the amplification of genes/sequences of interest; Table S2. Predition of transcription terminator on the phage genome; Table S3. ORF analysis of the phage genome; Figure S1. HindIII, EcoRI and SalI restriction patterns of the linear miniplasmid pJB011 determined by in vitro (A) and in silico (B) analysis.
