# Supplementary material for: The Novel Yersinia enterocolitica Telomere Phage vB_YenS_P840 Is Closely Related to PY54, but Reveals Some Striking Differences
Source: Viruses. 2023 Sep 28;15(10):2019. doi: 10.3390/v15102019 (PMC10612081; doi:10.3390/v15102019)
Supplement: Supplementary file 1 [file viruses-15-02019-s001.zip › Supplemental Material Figure S1.pdf]

Supplemental Material Figure S1.

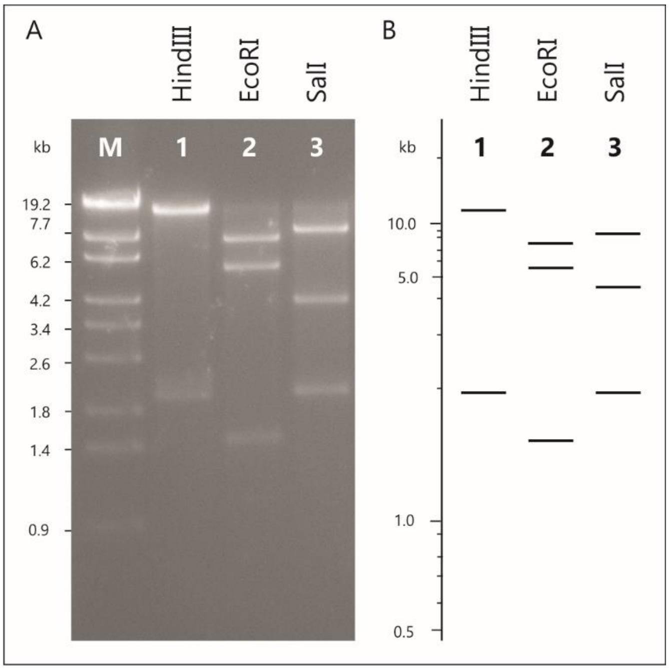

Figure S1. HindIII, EcoRI and SalI restriction patterns of the linear miniplasmid pJB011 determined by in vitro (A) and in silico (B) analysis. In silico restriction analysis and visualization was conducted using NEBcutter v 2.0 (http://nc2.neb.com/NEBcutter2/index.php; access date: 2023-03-08)
